# Transition experiences of Middle Eastern midwives into Australian practice: A multiple case narrative study

**DOI:** 10.1111/jan.15531

**Published:** 2022-12-13

**Authors:** Kolsoom Safari, Lisa McKenna, Jenny Davis

**Affiliations:** ^1^ School of Nursing and Midwifery La Trobe University Bundoora Victoria Australia

**Keywords:** Australia, immigrants, internationally qualified healthcare professionals, Middle East, midwifery, nursing, transitional experiences

## Abstract

**Aim:**

To explore transition experiences of Middle Eastern qualified midwives into practice in Australia.

**Design:**

This was a qualitative study using multiple case narrative approach underpinned by structuration theory.

**Methods:**

A total of 19 Middle Eastern qualified midwives from different states of Australia participated in this study. Individual semi‐structured interviews were conducted between November 2020 and September 2021, digitally recorded and then transcribed. Transcriptions were analysed in three stages, with three main categories generated in the second stage and a core category that was developed in third.

**Results:**

Entering the Australian workforce, Middle Eastern qualified midwives had to reframe their professional identities to fit the new system by adjusting to three aspects of the practice, including *preparation for practice, scope of practice and context of practice*. While they were prepared by medically oriented curricula, worked in systems that had limitations for midwives to practise in antenatal and postnatal and lacked regulation standards, they learned to practise autonomously in their full scope in a standardized context in Australia.

**Conclusion:**

Middle Eastern qualified midwives in Australia re‐evaluated their practice in their home countries, realized the gaps and adjusted to new ways of practising in Australia.

**Impact:**

To effectively use the potential of Middle Eastern midwives for workforce sustainability in Australia, support should be available to enable them to develop the necessary competencies for safe practice in Australia including provision of context‐specific transition programmes prior to registration and supporting mentorship after their integration into the Australian healthcare workforce.

**Patient or public contribution:**

Patient or public contribution does not apply to this study as its purpose was to explore the transition experiences of Middle Eastern qualified midwives themselves.

## 1 INTRODUCTION

### 1.1 Background

Many developed countries, including Australia, rely on international healthcare workers to accommodate for a shortage of local staff and rising population healthcare demand (Aluttis et al., 2014; Safari et al., 2022). The shortage of midwives in Australia caused by an ageing workforce, insufficient recruitment and retention rates has resulted in the decline and fragmentation of childbirth and maternity health services, particularly in remote areas of the country (Hildingsson et al., 2016). With existing and anticipated critical shortages of midwifery workforce by 2030, Australia is expected to continue relying on internationally qualified midwives to mitigate workforce sustainability issues (Department of Health, 2019).

Australia is a country with a diverse and growing migrant population from 190 countries, with 29.8% of the Australian population born abroad (Australian Bureau of Statistics, 2021; Booth & Tickle, 2003). Women who experience difficulty with the host country language or culture are prone to problems accessing services and are at risk of mental health problems (Heslehurst et al., 2018). Australian health system faces a significant challenge in providing accessible and high‐quality care for migrant women (Mander & Miller, 2016). Inclusion of culturally diversified personnel in healthcare was found to be a lifesaving strategy for providing culturally and ethnically diverse care (Sherwood & Shaffer, 2014).

Migrating to Australia to work as an internationally qualified midwife can be challenging as one must go through rigorous assessment processes to meet strict qualification standards, English language proficiency and professional competence to determine whether they have the necessary knowledge, skills and professional attributes needed to practise in Australia (Nursing and Midwifery Board of Australia, 2020). Australia's registration regulations for internationally qualified midwives seeking professional registration in Australia have evolved significantly over the past two decades, and standards introduced for internationally qualified midwives from non‐English speaking backgrounds have become progressively more complex (Nursing and Midwifery Board of Australia, 2011, 2014, 2020). Internationally qualified healthcare professionals from non‐English speaking backgrounds in developed countries have been found to be more vulnerable and experience more challenges even after registration than their counterparts from English‐speaking backgrounds in the host countries (Charlesworth & Isherwood, 2021; Safari et al., 2022).

It has been evidenced that migration of healthcare professionals from non‐English speaking countries, has reportedly grown recently to address shortages of local personnel and rising population healthcare demand in developed countries(Safari et al., 2022). Middle Eastern countries are among those from which refugee and skilled migration to Australia, the United States and Canada has surged significantly in the last decade, particularly due to political and socioeconomic instability in the region (Hatton, 2020; Safari et al., 2022).

Midwifery in Middle Eastern countries has developed dramatically in the recent years and progress is being made towards achieving International Confederation of Midwives (ICM) standards of practice, education and regulation (Safari et al., 2021). Over the past two decades, midwifery education in Middle Eastern countries has progressively moved into institutions of higher learning, and baccalaureate degrees in countries like Iran, Jordan, Palestine and Lebanon are being adopted for entry into practice. Iran, Iraq, Israel, Jordan, Lebanon, Morocco, Saudi Arabia, Tunisia and UAE are among countries that have membership with ICM (Safari et al., 2021). The majority of Middle East countries have legislation that recognizes midwifery as an autonomous profession, excluding Djibouti, Oman, Somalia and Yemen, and registration with regulatory authorities is mandatory for practice (Safari et al., 2021).

While published literature focuses primarily on the transition experiences of internationally qualified nurses and physicians, a few have examined the transition experiences of other disciplines, with only one study on midwives out of 48 studies included in the narrative scoping review conducted by Safari et al. (2022). Additionally, the literature appears to have frequently included midwives in the context of nurse migration (Javanmard et al., 2017). The only study conducted on internationally qualified midwives in Australia was a qualitative descriptive study performed by Javanmard et al. (2020) to investigate the transition experience of midwives primarily from English‐speaking countries with a similar midwifery context to that of Australia, such as the United Kingdom and South Africa. In addition, the influence of overseas experience of international midwives on their transition to the Australian midwifery system was not investigated in this study (Javanmard et al., 2020). However, midwifery practice in Middle Eastern countries has been found to be significantly different from that in developed countries, which would affect the transition experience of midwives in host countries (Batinelli et al., 2022; Safari et al., 2022).

With existing and anticipated critical shortages of midwifery workforce, Australia will continue relying on internationally qualified midwives to mitigate workforce sustainability issues. Successful integration of midwives from Middle Eastern countries into the Australian health system has the potential to make a substantial contribution, not only to the sustainability of the midwifery workforce in Australia but also to expansion of diversity in the system and promotion of culturally competent maternity care. However, little is known about challenges experienced by midwives from these countries, and more generally non‐English speaking backgrounds, in Australia and other developed countries relying on internationally qualified midwives. Thus, this study aimed to examine the transition experiences of internationally qualified Middle Eastern midwives into practice in Australia informed by the following research questions:

- What are the transition experiences of the Middle Eastern qualified midwives in Australia?
- How their overseas midwifery experiences impacted their transition into practice in Australia?

### 1.2 Design

A multiple case narrative approach was used to capture Middle Eastern qualified midwives' dialogue to understand their transition experiences and the meanings they made of their experiences, by enabling them to construct and articulate their stories (Shkedi, 2005). Multiple case narrative, developed by Shkedi (2005), is a constructivist–narrative approach that borrows some elements from grounded theory (Shkedi, 2005). It collects information on a wide range of cases narratives, treats them as clusters of categories and promotes broad cross‐case analysis for generalization purposes using an associational approach (Shkedi, 2005).

While evidence suggests that integrating immigrant healthcare professionals into the professions is influenced by socially constructed characteristics, research on the social structures determining workforce integration is limited (Xiao et al., 2014). As defined by Giddens, social structure refers to the rules and resources that are either formal (policies) or informal (tactical and cultural norms). Social structures and human behaviours (or agency) do not exist in isolation as a ‘dualism’, but as a ‘duality’, inextricably linked and affected by one another (Giddens, 1984, p. 25). All systems are reconstituted by structures: sets of ideas and principles that govern the practices of individual agents. Structures are virtual in nature, existing as memory traces that inform the practices of knowledgeable agents. An agent acting in a system will learn how to go on in accordance with the appropriate structures (McGarry, 2016).

The multiple case narrative approach developed by Shkedi (2005) underpinned by Gidden's structuration theory (Giddens, 1984), provided a practical technique for examining transition experiences of Middle Eastern midwives to midwifery practice in Australia.

### 1.3 Participants

Internationally qualified midwives were included in the study if they: (1) migrated to Australia; (2) were Middle Eastern educated midwives; and (3) had professional experience in Middle Eastern countries. A Middle Eastern qualified midwife was defined as a midwife who received their first qualification to practise as a midwife in a Middle Eastern country.

### 1.4 Data collection

Participants were recruited nationally from Australia between November 2020 and September 2021, mainly through advertising via the Australian College of Midwives (ACM) and snowball sampling. In addition, local Arabic and Muslim groups in Australia were approached and provided with details for the study and asked to disseminate invitation emails to their memberships and place a short recruitment notice on their social media platforms. When Middle Eastern qualified midwives with an interest in the study contacted the researcher, they were sent an email and asked to review the participant information form provided to ensure that they were well informed about the study and eligibility requirements for participation. If they met the eligibility criteria and agreed to participate, a convenient time for telephone or zoom interview was arranged with them. Although, completing and returning the consent form by participants was taken as indication of their agreement to participate in this study, they were asked to verbally confirm that they were still willing to participate in the study prior to the interview commencing.

Semi‐structured interviews were conducted in English with 19 participants by the primary researcher (six telephone and 13 online interviews via zoom). Semi‐structured interviews were chosen as they offer continuity of questioning informed by the literature, while remaining open to new topics which can be introduced by participants (Wahyuni, 2012). To confirm the scope and relevancy of the content of the preliminary guide, establish the potential need to reformulate questions and evaluate its execution, the interview guide was pilot tested using an internal testing technique (Kallio et al., 2016). Internal testing referred to the collaborative review of the preliminary interview guide by the research team's investigators (Barriball & While, 1993). This approach could provide essential information about the interview guide in particular, including the elimination of ambiguities and improper leading questions and the identification of any potential bias (Chenail, 2011). In this study, the primary investigator adopted the role of the participant and was interviewed by another researcher. Gaining insight into how it felt to be interviewed encouraged the ethical and responsible manner in which sensitive patients were addressed (Kallio et al., 2016).

Interviews lasted an average of 1 h and ranged between 40 and 150 min. The interview guide focused on the professional integration experiences of participating Middle Eastern qualified midwives, following the sequential migration process from their pre‐arrival experiences through the challenges they encountered with registration and practice after arriving in Australia. The interview questions were mostly drawn from a previous scoping review conducted to inform the broader study (Safari et al., 2022). Each interview dialogue began with one broad, open‐ended question to initiate storied responses, such as ‘What have you been doing since you arrived in Australia?’ As each interview unfolded, follow‐up questions were asked, encouraging continuous narratives, and further investigating emerging themes. The participants' overseas practice and education experiences were also explored during the interviews, as it was found to be relevant to their transition experiences in Australia (Safari et al., 2022). These questions were developed in accordance with the International Confederation of Midwives competency standards for practice, education and regulation (Butler et al., 2018; Thompson et al., 2011). Table 1 contains examples of interview questions used for exploring the transition experiences of Middle Eastern qualified midwives into practice in Australia.

**TABLE 1:** Interview questions

What are the requirements for studying in a midwifery programme in your home country?
2 What are the available degrees for midwives to study in your home country?
3 What midwifery qualification is required for entry into practice in your home country?
4 How was the midwifery curriculum in your country? Did your midwifery programme include both clinical and theoretical components?
5 What were the requirements for graduation?
6 What was the qualification of midwifery teachers in your home country?
7 How would you compare midwifery education in your home country to Australia?
8 Did you apply for professional registration in Australia?
9 What were the requirements for your registration?
10 Are you currently practising midwifery in Australia?
11 Did you have orientation into practice in Australia?
12 In which clinical area have you been practising midwifery in Australia? How did you find it to be different from your home country?
13 Do you have the autonomy to practice midwifery in the full scope in Australia? How did you find it to be different from your home country?
14 Do you practice updated midwifery practice standards in Australia? How did you find it to be different from your home country?
15 Do you find midwifery practice in Australia to be legally oriented? How did you find it to be different from your home country?
16 How was your experience with documentation in Australia? How did you find it to be different from your home country?
17 How would you compare midwifery practice in your home country to Australia?
18 What are your main challenges with midwifery practice in Australia?

Data collection was completed after 19 interviews, when thematic saturation was deemed to have been achieved (Guest et al., 2006; Shkedi, 2005).

### 1.5 Ethical considerations

The research received ethics approval from the La Trobe University Human Ethics Committee (Approval Number: HEC20403). Confidentiality, informed consent, freedom to refuse to participate or withdraw from the study, and refusal to discuss specific issues were addressed at all stages of the research project. The data were deidentified and securely stored in accordance with the National Statement on Ethical Conduct in Human Research, Australia (National Health and Medical Research Council, 2015).

### 1.6 Data analysis

Interview transcriptions were uploaded to NVivo 12 and analysed in three stages as recommended in the multiple case narrative approach (Shkedi, 2005). At initial stage, the data were segmented, allowing for the identification of categories without requiring consistency or a connection between categories to be apparent in NVivo 12, which facilitates the management of the vast amount of information that the researcher must deal with. The process of grouping together components of data that pertain to the same phenomenon is referred to by Shkedi as categorization (Shkedi, 2005). This starts with a few broad categories to establish a general overview and then finding ways for progressively refining the data. In the mapping stage, families of categories were beginning to emerge as cross‐case analysis of emergent categories and their prevalence was explored, which resulted in finding relationship between pattern of main categories in different groups of participants.

Focused categorization occurs when the researcher centralizes data into a cohesive account around the core categories and starts to formulate a better picture of study findings (Shkedi, 2005). Focused analysis continued until the writing up of the findings, where core categories were created. In the process of producing core categories, the researcher sought out the category that had the ability to generate a coherent narrative, what appeared to be the key focus or issue of the participants, was frequently present in the data, and was easily related to other categories.

Three research team members conducted analysis by contributing to the development of primary categories in the second stage and core category in the third stage. Throughout the analysis process, the team reviewed interview experiences and discussed findings in weekly meetings. The emergent themes were reviewed several times until consensus was reached. The involvement of the research team's reflections and constant comparative method enabled generation of credible conclusions. Structuration theory used in this study informed analysis of the impacts that Australian practice standards and regulations had on the way in which Middle Eastern qualified midwives practised their profession in Australia, as well as strategies they employed to adapt to challenges they encountered during this process.

### 1.7 Rigour

The first author conducted preliminary data analysis, communicated findings to the team for cross‐checking and led regular team meetings to discuss findings. To address dependability, an audit trail was created storing all codes and coded data in a matrix in NVivo 12 (Shkedi, 2005). This allowed for reviewing, verifying and auditing of the coding schema and associated data by the co‐authors prior to finalizing the analysis. Trustworthiness and rigour were further addressed through methods of reflexivity (Shkedi, 2005). The primary researcher, who is an internationally qualified midwife, undertook the analysis in a reflexive manner, in which personal beliefs, assumptions and roles were continually discussed in the team during analysis to prevent premature interpretations of the data and recognize any assumptions and bias (Malterud, 2001).

Respondent validation, the process of researchers discussing interpretations of data with study participants, who may verify, alter and offer feedback as to whether they are recognizable and compatible with their experience also contributed to the elimination of bias in this study (Roberts & Priest, 2006). Thus, whenever the validity of the researcher's interpretation was doubted, participants were contacted and requested to check their transcribed and interpreted data and reject or amend any components with which they disagreed. Reporting in this manuscript is in accordance with COREQ guidelines (Tong et al., 2007).

## 2 FINDINGS

### 2.1 General characteristics of the participants

Nineteen Middle Eastern qualified midwives participated in this study; general characteristics are presented in Table 2. Their average age was 44 years, 17 participants (90%) were Iranian and spoke Farsi as their first language, while the remaining 10% (*n* = 2) were Lebanese who reported Arabic as their first language and French as their second. The majority (43%) resided in the state of New South Wales, while others were living in other states/territories including Victoria, Queensland and the Australian Capital Territory. Most participants had migrated to Australia via student (37%), dependent skilled worker (26.5%) and partner visas (26.5%). All participants held a bachelor's degree in midwifery from Iran or Lebanon; seven also had a master's degree from Iran; and six studied PhD degrees in Australia. Average work experience among participants was 7 years overseas and 8 years in Australia. The majority of participants working clinically, were working in postnatal area in Australia, having no previous postnatal experience overseas. A significant proportion worked in the fields of research and education, both overseas (36.5%) and in Australia (30%).

**TABLE 2:** General characteristics of the participants (*n* = 19)

Participants' characteristics	Frequency
Age (years)	
Average	44 ± 8
Range	28–60
Nationality	
Iran	17 (90%)
Lebanon	2 (10%)
First speaking language	
Farsi	17 (90%)
Arabic	2 (10%)
Residential state/territory in Australia	
New South Wales	8 (43%)
Victoria	6 (32%)
Queensland	3 (15%)
Australian Capital Territory	2 (10%)
Immigration visa type	
Student visa	7 (37%)
Dependent skilled worker visa	5 (26.5%)
Partner visa	5 (26.5%)
Refugee	1 (5%)
Work and holiday visa	1 (5%)
Overseas Education	
Bachelor's degree in Midwifery (Iran, Lebanon)	12 (65%)
Master's degree in Midwifery (Iran)	7 (35%)
Australian education	
PhD	6 (32%)
Masters degree in midwifery	2 (10%)
No degree	11 (58%)
Overseas working experience (years)	
Average	7 ± 6
Range	1–21
Sector of working overseas	
Labour and birth	7 (35%)
Antenatal	5 (26.5%)
Education and research	7 (36.5%)
Working experience in Australia (years)	
Average	8 ± 6
Range	2–20
Sector of working in Australia	
Postpartum	7 (35%)
Labour and birth	2 (10%)
Education and research	6 (30%)
Assistant midwife^a^	1 (5%)
Pathology collector	1 (5%)
Disability worker	1 (5%)
Not working	1 (5%)

a Working under direction of the registered midwife to deliver limited care to women.

### 2.2 Second stage of analysis

In accordance with the multiple case narrative approach, following analysis of individual cases in the first stage, cross‐case analysis was performed on all narratives in the second stage, and the professional transition experiences of Middle Eastern qualified midwives were classified into three categories, including *preparation for practice, scope of practice and context of practice*.

#### 2.2.1 Preparation for practice

Participants described characteristics of midwifery education programmes in Middle Eastern countries including admission requirements, programme duration, available midwifery degrees and clinical and theoretical components that prepared midwives for practice. Education system characteristics in Iran and Lebanon were generally similar. Entry requirements for midwifery programmes included 12 years of schooling and successfully passing the national entrance exam in both Iran and Lebanon (P3, P4, P6, P15, P17 and P18).

A bachelor's degree in midwifery was required for practice in these countries, which was obtained through 4 years of university education via direct entry (P6, P16 and P10). Postgraduate degrees including master's degree and doctorate (PhD) programmes were available to study for midwives in Iran, but not in Lebanon (P2, P18, P3, P9). The midwifery curriculum consisted of both clinical and theoretical components in Iran and Lebanon. According to Lebanese midwives, theoretical participants were totally taught by obstetricians in that country (P4, P6). Iranian participants also stated that obstetricians lectured into midwifery participants in this country and that they used obstetrics textbooks, rather than midwifery sources to study (P2, P3, P5, P8, P9, P16, P18 and P14).

Clinical components of courses were described as focusing on instruction of foundational midwifery skills in the first year, becoming more specific and increasing in length towards the last year (P6, P19). Clinical placements for midwifery students were carried out in a variety of settings, including prenatal, labour and delivery and family planning settings in these countries (P8, P18, P4, P2, P11 and P15).

Graduation criteria for the midwifery students in Iran included conducting 80 births and 40 episiotomy suturing.

…I remember I needed to have at least 80 deliveries, 40 of those births needed to be primi mothers to suture their episiotomy… I needed to be the main midwife managing the birth, but of course supervised by my teacher. (P2)

The requirements for graduation in Lebanon were performing 55 deliveries and suturing 10 episiotomies.

In order to be graduated, you should do at least 55 deliveries. And 10 of them were with episiotomy. You should do suturing… (P4)

A majority of participants in this study also experienced the midwifery education system in Australia by working in academia (P2, P3, P8, P9, P11, P15 and P18) or studying midwifery programmes as they were required to upgrade their overseas qualifications for registration (P5, P7, P12, P17 and P19). Therefore, they often compared the midwifery education programmes in their home countries with those in Australia. Some appreciated the broader curriculum employed in Middle Eastern nations, while others argued that the midwifery education in their home country was medicalized and featured more irrelevant participants compared with the midwifery‐focused curriculum offered in Australia (P3, P12 and P17).

In Iran, we study midwifery for four years, learning many aspects, not only midwifery like irrelevant subjects, like family planning, or treatment of vaginitis, all those things that midwife can't deal with. But in Australia, you learn to focus more in helping women to have baby and supporting them throughout the normal pregnancy. (P17)

They verbalized being impressed by the subject of midwife‐led continuity of care incorporated in midwifery curricula in Australia, in which the midwife as a principal care provider follows a woman throughout pregnancy, birth and postpartum periods (P17, P5).

Another great thing that I learned here is continuity of care, which we didn't have in Iran, so in here, like during my study at university, I had 10 women, I followed them up all by myself throughout pregnancy, during all that antenatal care, and I was them during birth…so, it was new thing for me. (P17)

#### 2.2.2 Scope of practice

Participants perceived the midwifery scope of practice in Middle Eastern countries to be very different from that in Australia. Iranian graduate midwives identified that the scope of midwifery practice in Australia was more limited than in Iran, where their role was similar to that of obstetricians and gynaecologists (P7, P8, P9, P10, P12, P13, P14 and P18). They indicated that midwives in Iran were authorized to practice in their own private clinics in that country. They were also eligible to independently provide family planning methods and administer certain medications and diagnostic tests for common vaginal infections and pregnancy (P2, P8, P11 and P18).

…I remember in Iran; midwives can do more than the Australian midwives. For example, when I was working in Iran in my clinic, I could do family planning; inserting IUD [intrauterine device], prescribing oral contraceptive pills or other types of contraception methods, but in here I think the maternity child health nurses, they can do that, but not the midwife. …I can say we were probably similar to maybe obstetrician or gynecologist in Iran, with limited practice. (P13)

Some had opposing views and argued that midwives in Iran undertook responsibilities that were not for them, whereas Australian midwives largely focused on skills that fell in their scope (P3, P17),

Midwifery in Iran is a mixture of everything, women health, midwifery, nursing, and family planning, it is not a midwifery job. Even I don't think that midwives can take pap smear here; nurses can take it, but midwives cannot. But they are very strong in labour and delivery care. (P3)

They mentioned that antenatal care was predominantly delivered by obstetricians in their home countries as women preferred them over midwives (P3, P4, P6 and P16).

Antenatal care is not provided by midwives in Lebanon. A pregnant woman likes to go to a specialist, to obstetrician… (P4)

Both Iranian and Lebanese midwives highlighted that they had never worked in postpartum wards in their home countries because, in Iran, this care was routinely provided by nurses and, in Lebanon, women were discharged from the hospital shortly after giving birth in Lebanon, so there was no time for postpartum care. This experience made it challenging for them to be expected to provide postpartum care in Australia. (P1, P10 and P15)

It's a whole different system in Lebanon. Like, we didn't have postnatal care in Lebanon. The woman would have her baby, then go home… after couple of hours and there's no follow‐up because the woman goes home so early. Here, no, they stay, and we take care of them… it is a bit challenging …(P6)

Mothers' personal care and breastfeeding education following delivery were routinely provided by family members in Lebanon, and women relied more on their relatives than midwives. Thus, delivery of these forms of care in Australia were perceived as unfamiliar and an additional burden for Lebanese midwives.

In Lebanon, it's the aunty and the mother support mother. They can give advice about how to breastfeed and attach and all these things and if it doesn't work, they go to formula. They rely more on family than the midwives. Here, it does put more pressure on the midwife to help more and support – bathing and feeding and dressing of mother, all these little things that the family would have helped. (P6)

Similar experiences were reported by Iranian midwives. They highlighted that, unlike Australia where midwives are expected to assist women with personal care in postpartum wards, cleaners, enrolled nurses or family members of the women routinely undertook these roles in Iran.

In Iran we wouldn't do lots of thing as a midwife. Cleaner would have done it, the enrolled nurse or husband or mum or sister would have done it. In here, we have to do everything, which I agree with that. Like, for example, if patient going to have ‐ in postnatal‐ have a shower, lots of Iranian midwife were offended if you tell them, “You need to get patient up and take her ‐ help her with having shower”, but here, midwives should do everything. (P15)

Providing labour pain management, particularly using epidural anaesthesia, was another new aspect of midwifery in Australia for participants as it was not practised it in their home country, so they reported lacking the relevant skills (P1, P2, P10, P12 and P18).

… in Lebanon there's no, back then there was no focus on pain relief. We weren't taught much about pain relief, so the women would just stay in bed the whole time. … So, when I came here, one of the tests for registration I had to do was about pain relief, and I failed that test …, lots of women using epidural here… looking after them needs different training. (P6)

Practising midwifery model of continuity of care in Australia, which was not part of midwives' roles in Middle Eastern countries, was described by participants as ‘wonderful’ (P17), ‘absolutely excellent care’ (P13) and ‘interesting care’ (P19) that ‘makes a lot of difference by building trusting relationship with women’ (P5).

Moving to a new model of midwifery care in Australia that places women at the centre of care and involves them in decision‐making was reported as a new and satisfying experience for participants. They referenced their overseas practice, where midwives made the majority of decisions and women were not consulted for their consent about the care they received (P2, P4, P6, P7, P9, P11, P12, P15, P17 and P18).

… here they really appreciate the women, and their body and their choice, which in Iran they don't appreciate the women choice at all. The way they treat patients in Iran is terrible and here they just respect women and what they decide and what they do. Well, you can't do anything without their permission, in Iran midwives force women to do what they wanna do, so here is a lot different in ways that respect women better. (P19)

However, providing women‐centered care was found demanding by some participants (P1, P6) as accommodating all the labouring woman's needs was challenging and they believed that mothers’ decisions were often not medically appropriate. One pointed to her experience working overseas, where she provided intrapartum care for a couple of women, which she found more manageable than delivering one‐on‐one care in Australia.

In Lebanon we had to take care of more than 10 patients in labour, but, still it was easy… here, it is one‐to‐one care, one midwife for one patient. It was difficult for me, as mothers have many choices, sometimes she is in pain and she does not want to use epidural, and she just want gas, so you give her gas, and she will use the gas for some time, and again she thinks the gas is not helping her with her pain, so she asks for epidural…you know it is a lot of work for me in the labour room…sometimes you think you should give a care to the patient, as you think it is right for her based on your knowledge, but she does not want that, and she deny your care, so you don't know what to do. (P6)

According to participants, midwifery practice in Iran and Lebanon was interventional and not evidence‐based. They stated that women in these countries were not given sufficient support during labour and that their labours were commonly expedited by any form of intervention, including induction of labour and routine episiotomy (P3, P6, P7, P9, P18, P15 and P17).

We do not allow patient to have normal delivery process, we're doing something like injections, anything as long as we make the delivery process shorter…we normally do episiotomies for all the primi [primigravidas]… But in Australia no, we just support the patient to have a normal birth. (P6)

#### 2.2.3 Context of practice

When comparing the systems, participants from Lebanon frequently mentioned that they had limited autonomy and independence over their practice in Iran and Lebanon (P3, P6, P10, P9, P16, P17, P18 and P19). Midwives’ professional status was also seen to be more valued in Australia, where they worked autonomously as part of a multidisciplinary team and were enabled to practice in their scope (P3, P9, P16 and P19).

I just feel sorry for the midwives back home. Here, they [healthcare team members] really appreciate what the midwives do, like we work along with the obstetrician and pediatricians, and no‐one interfere with others, and they work in a multi‐disciplinary team. (P19)

Lebanese midwives indicated that all the legal responsibilities of maternal care was with obstetricians, and that obstetricians were penalized for midwifery misconduct in Lebanon (P4, P6). Participants from both countries discussed their new experiences in Australia that necessitated legally conscious midwifery practice.

In Lebanon, obstetricians are responsible for whatever we do… in Australia, if you give the patient a Panadol tablet wrong, the patient can take her right directly. But you should be very very careful about each task you are given. (P4)

Following strict documentation rules and responsibilities for midwifery practice in Australia was discovered to be a significant difference between Australia and participants’ home countries. While documentation was not practised or was quite restricted in Middle Eastern countries, it was an essential component of midwifery practice in Australia, and midwives were expected to record details of care provided (P5, P6, P11, P12 and P15).

I always was told that you've got to be very careful, make sure you always document, because one day, there will be one day when you're going to be called to court to be a witness. I see that they have written “I gave her a bottle of water” or “I turned the light off” or “I draw the curtain” or like similar, very simple things like this, they document everything. When I was in Lebanon, there was no – we hardly ever documented. There was no documentation. (P6)

Australia's midwifery practice was differentiated from that of Middle Eastern countries by its strict adherence to clinical guidelines and clear and precise job descriptions (P1, P2, P4, P5, P12, P17 and P19).

There were not clear protocols in Iran. Here, we have our own protocols which came from the head organisation. And we are definitely needed to be practising based on those protocols. (P2)

While participants realized that adhering to regulation standards and strict documentation in Australia contributed to increasing the safety of midwifery practice, they were frustrated as they were not familiar with this model of practice in their own country. They also indicated that because a major portion of their time was spent on documentation in Australia, they were unable to deliver midwifery care that they used to do in their home country. According to participants, abiding to Australia's stringent regulations decreased their confidence to the point where they reported deciding not to practice some skills to avoid legal repercussions (P5, P, P11 and P15).

I think some of roles in Australia are good, some of them are too much. It exhausts you; you know it just puts lots of stress on you, but in a sense that they think about every detail, it's good for staff safety… Since I came, I haven't done stitching because of the paperwork and extra work I already have to deal with in my shift, …I just call doctor to come and do it. (P5)

They found it unusual and restricting to be required to obtain further training and certificates to practice some skills in Australia that they had regularly undertaken after graduation in their home countries (P6, P12, P13, P15 and P19).

For us was very funny to have to go and do a course to be able to take blood, because it was part of everybody's job in Iran. But in here, everything, you should have a certification ‐ you should go through a course and be certified to be able to do that. (P15)

They found the Continuing Professional Development (CPD) programme and its rules in Australia to be well organized and effective for updating their skills, in contrast to the situation in Lebanon or Iran, unlike in their home countries, where it was either unavailable (Lebanon) or very limited (Iran) (P1, P4, P8, P9, P11, P15 and P17).

Here, every year you have to have at least 20 CPD points…for those who work, they have to do – every year they have to do training modules on all aspects of labour and birth, like the dystocia, postpartum haemorrhage and … So, you have to do 20 hours, so it could be in any of these. So, it's really good system. (P11)

### 2.3 Third stage of analysis

The third stage of analysis was devoted to methodically combining the generated categories into a core category of *reframing professional identity*. According to the case narratives in different categories of *preparation for practice, scope of practice* and *context of practice* collectively, Middle Eastern qualified midwives modified their practice to fit Australian standards, and in so doing modified their professional identities. They re‐evaluated the practice of their home countries, adjusted to new ways of practising in Australia and acquired new sets of responsibilities and professional relationships as part of the process of restructuring professional identity.

When the characteristics of midwifery education in Middle Eastern countries were reconsidered, participants discovered inadequate preparation to practice according to Australian standards, as their curriculum lacked a midwifery focus by inclusion of obstetricians as main educators and that did not support midwifery concepts. Additionally, while the Australian Health Practitioner Regulation Agency (AHPRA) maintains strict partnerships with universities and precisely defines the criteria expected from midwifery graduates, citation of inadequate collaboration between regulatory organizations and education providers about requirements for midwifery students demonstrated lack of education standards in home countries and accreditation of education programmes in these countries.

… here the AHPRA is very connected to universities, they say if you don't do this and this, we don't approve your graduates, you know, they need the approval of AHPRA. They say if your students don't get this amount of practice or these assignments, we don't give them qualifications. But in Lebanon, there is no link and universities are independent. (P6)

The scope of practice, and the authority that they held in the workplace in Australia, were all a novel experience for them. Participants reported having to upskill and develop new competencies to be able to practice in full scope of Australian midwifery practice as they lacked overseas working experience in prenatal and postnatal care.

Participants described stripping themselves of their previous professional identities and transition to Australia from Middle Eastern countries where midwives' autonomy was restricted due to interprofessional power struggles among physicians and midwives. Practising autonomously in well‐defined job boundaries in Australia, they did not have to work beyond their scope of practice, while there were unclear boundaries between their own and obstetricians' roles in Middle Eastern countries, where midwives undertook responsibilities that were for obstetricians (P4, P9, P10, P17, P18 and P19).

It's much easier here, much easier, not so much work…in Lebanon, you should do everything, we are alone in labour, and do whatever doctors do … here, they have well‐defined job description. They only work within their scope, if there is something they don't know, they refer it to the obstetrician. (P4)

Middle Eastern midwives, who were not legally accountable in their home countries, adopted new professional identities that conformed to documentation norms and evidence‐based guidelines in transition to the litigation‐conscious midwifery practice prominent in Australia. Previously, they had followed obstetricians’ instructions as there were no clinical protocols accessible and obstetricians determined clinical rules as the dominating profession in these countries (P1, P2, P5, P12, P17 and P19).

Back home is mostly what the obstetrician decides to do, it is not what the guideline says. Actually, there were not clear protocols in Iran, it is what the obstetrician wants to do…. midwives have to do obstetrician's order… I just felt that in Australia everything is based on guideline, and the midwives really stick to them … (P19)

## 3 DISCUSSION

Middle Eastern qualified midwives came to Australia with already well‐formed professional identities reflecting their overseas context of practice; thus, entering the Australian work system they had to adjust their imported identities as professionals by revaluating the familiar patterns of skills, re‐learning the role of their professional cadre in the workplace and adapting to the way their profession was practised in Australia. To the author's knowledge, this is the first time that the transition experiences of non‐English speaking background midwives from countries with midwifery education, practice and regulation that differ significantly from that of their host country is reported. The limited literature on internationally qualified midwives has been primarily focused on midwives who are coming from a similar system that was as developed as their host countries (Javanmard et al., 2020). Thus, the issues reported by participants in this study were found to be significantly different, with more focus on how considering the remarkable gaps in their overseas practice, Middle Eastern midwives adjusted to the new way of practising that required them to adhere to high professional standards.

Middle Eastern midwives participated in this study had been in practice for several years prior to migrating to Australia and had gained entry into Australia, demonstrating that their qualifications and skills met Australian standards. Thus, modifying their professional identities to fulfil Australian standards of practice required them to strike a balance between presenting themselves as qualified to practice in Australia and determining how they needed to adapt their professional presentation to fit in. A similar observation was found in a Canadian study, where the process of professional adjustment was more profound for international health professionals from non‐western healthcare systems, than for their counterparts from countries with comparable systems to Canada such as the United States, United Kingdom, Australia and France (Neiterman & Bourgeault, 2015).

When comparing the characteristics of their previous training and education to Australian standards, Middle Eastern midwives discovered important gaps in the way they were prepared for practice as their midwifery curriculum was not standardized, medically oriented and primarily lectured by obstetricians. Coming from medicalized systems was also evident in the terminology they used in interviews: ‘patient’ instead of ‘woman’ and ‘delivery’ rather than ‘birth’. In Australia, regulatory authorities implement accreditation standards for developing midwifery curriculum that are guided by ICM standards for midwifery education (ICM, 2014), which emphasize the philosophy that introduces and prepares midwives as advocates for non‐intervention childbirth and are primarily educated by qualified midwifery lecturers (Hainsworth et al., 2021). Middle Eastern qualified midwives must be properly assessed against the education standards expected from graduate midwives in Australia before registration, and opportunities to upgrade their qualifications should be made available to compensate for gaps in their overseas education (ANMAC, 2014).

Another aspect of midwifery care lacking in midwifery education and practice of Middle Eastern midwives and required significant professional adjustment was midwifery‐led continuity of care, which is included as a mandated clinical practice‐based learning component in midwifery programmes leading to registration in Australia from 2009 (Tierney et al., 2018). Midwife‐led models of care are viewed as an essential strategy for strengthening women's choice and a fundamental component of woman‐centred care in Australia (McLachlan et al., 2022), which was another feature of care that Middle Eastern midwives who participated in this study had not been exposed to prior to moving to Australia. Their transition to the Australian healthcare system, therefore, was not simply a process of adaptation to a new workplace; it was often acquiring new competence to provide new approaches to midwifery care. Thus, specific instruction should be provided for overseas midwives prior to registering in Australia to prevent them from encountering further issues. The transition of internationally qualified healthcare professionals has been described as comprised of interconnected stages, and unresolved issues in the registration stage can result in emergence of serious issues in the workplace (Safari et al., 2022).

The process of restructuring professional identity for Middle Eastern midwives required them to modify their practice and develop insights into how the status of their profession in Australia differed from what they were accustomed to. In contrast to UK midwives, who reported restricted authority for practice in Australia (Javanmard et al., 2020), Middle Eastern midwives in this study experienced greater autonomy in Australia, in contrast to their home country experiences where midwives were subordinate to obstetricians who held a predominant role in the hierarchical structures of the healthcare system. Rank and status of the profession establish roles in the workplace (Neiterman & Bourgeault, 2015), hence Middle Eastern midwives may struggle to adjust their professional identities without informing of them about their role as independent primary care providers in Australia and the responsibility that came with this status.

To adapt to the local model of practice in Australia, midwives from the Middle East learnt the new professional landscape of the host country that involved independent care of women across all stages of childbearing. This was in contrast to their overseas experience, where they did not practice prenatal and postpartum care because obstetricians were the primary antenatal care providers, and postpartum care was either not offered or provided by nurses. Additionally, they were required to support mothers with early postpartum self‐care, which was usually delegated to family members accompanying patients in their home countries. This was consistent with results of a scoping review that examined midwifery practice, education and regulation in Middle Eastern and North African countries in the context of ICM standards (Safari et al., 2021).Given that the majority of Middle Eastern midwives in this study worked in postpartum in Australia, indicating that they are more likely to be interested in or find employment in this area despite lacking overeseas experience, the question of how well they are prepared for this practice in Australia arises. Prior to registering Middle Eastern midwives, a context‐specific and evidence‐based upskilling programme, followed by mentorship customized to their needs in the workplace, is crucial to consider to maximize their proficiency and ensure safe practice and public safety in Australia.

Generally, learning which responsibilities are undertaken by midwives in the host country and which are delegated to other members of the healthcare team is considered a necessary first step towards professional integration (Neiterman & Bourgeault, 2015). Clear role description for midwives in Australia facilitated this transitional phase for Middle Eastern midwives and made practising in Australia easier for them, as they were overburdened by taking on obstetrics tasks because of the ambiguous division of responsibilities in their home countries.

Middle Eastern midwives, who were not legally accountable in their home countries, made significant adjustment to maintain legal practice by strictly following guidelines and documentation rules; however, for some it negatively influenced their confidence and discouraged them from practising skills that made them prone to litigation. Moreover, using additional time and attention to fulfil the increasing requirements of documentation towards regulatory standards interfered with practising other skills, such as episiotomy suturing, that they had normally practised in their home countries. Similarly, Australian midwives complained about having difficulty tailoring care to each woman due to the increasing amount of data that they were required to document (Verrall et al., 2015). Documentation requires specific skills for which Australian midwives are educated and prepared (Gray et al., 2019); it is important to recognize that Middle Eastern midwives may need more time to acquire it due to lack of overseas experience. Future studies may shed more insight on how delays caused by establishing new skills in the host country can impact international midwives' efficiency in performing other competencies that they were proficient in.

## 4 LIMITATIONS

Some limitations in relation to this study are acknowledged. Despite extensive recruitment strategies being employed, the participants do not represent the diversity of all Middle Eastern countries as they were predominantly from Iran. As evidence indicates, this could be related to the fact that the only degree available to midwives in some Middle Eastern countries is an associate degree, which is not eligible for registration in Australia (NMBA, 2020; Safari et al., 2021). Additionally, only Middle Eastern qualified midwives who felt confident in their experiences or language ability may have chosen to participate. Although the multiple case narrative research approach employed in this study has the potential for sample to population generalization, the sample size was insufficiently large and heterogenous for this purpose, yet, there are valuable insights from this work that may inform other health professions and policy contexts (Shkedi, 2005).

## 5 CONCLUSION

This study provides an opportunity to discuss views and experiences relating to the professional transition of Middle Eastern qualified midwives in the Australian context, an area that has been significantly under‐researched. Seeking professional integration in Australia, Middle Eastern midwives had to significantly modify their professional identities and learn new skills, roles and responsibilities to reflect a new practice context. The preparation, scope and context of midwifery practice in Middle Eastern nations were found to differ from those in Australia by lacking midwifery‐oriented curricula, autonomy to practice in full scope, clear job descriptions, evidence‐based guidelines and documentation rules. A significant gap in midwifery practice in Middle Eastern countries suggests that strategies should be developed to ensure their safe and women‐centred practice in Australia, including provision of context‐specific transition or upgrade programmes before registration, and supporting mentorship after integration into the Australian healthcare workforce. While healthcare systems of Australia, similar to the majority of the developed world will likely continue to rely on internationally qualified midwives, various stakeholders must acknowledge the level of support that Middle Eastern midwives require to practice competently and in accordance with Australian standards to maintain patient safety.

## AUTHOR CONTRIBUTIONS

Kolsoom Safari: Conceptualization, methodology, investigation, data curation and formal analysis, Writing: Original draft. Jenny Davis: Conceptualization, methodology, formal analysis, visualization, supervision, writing—review and editing and validation. Lisa McKenna: Conceptualization, methodology, formal analysis, visualization, supervision, writing—review and editing and validation.

## CONFLICT OF INTEREST

The authors declare no potential conflict of interest with respect to the research, authorship and/or publication of this article.

### PEER REVIEW

The peer review history for this article is available at https://publons.com/publon/10.1111/jan.15531.

## Data Availability

The data that support the findings of this study are available on request from the corresponding author. The data are not publicly available due to privacy or ethical restrictions.
